# Heat Stress Impacts on Lactating Cows Grazing Australian Summer Pastures on an Automatic Robotic Dairy

**DOI:** 10.3390/ani10050869

**Published:** 2020-05-17

**Authors:** Richard Osei-Amponsah, Frank R. Dunshea, Brian J. Leury, Long Cheng, Brendan Cullen, Aleena Joy, Archana Abhijith, Michael H. Zhang, Surinder S. Chauhan

**Affiliations:** 1 School of Agriculture and Food, Faculty of Veterinary and Agricultural Science, The University of Melbourne, Parkville, Melbourne, VIC 3010, Australia; ROsei-Amponsah@ug.edu.gh (R.O.-A.); fdunshea@unimelb.edu.au (F.R.D.); brianjl@unimelb.edu.au (B.J.L.); long.cheng@unimelb.edu.au (L.C.); bcullen@unimelb.edu.au (B.C.); aleenajoyj@student.unimelb.edu.au (A.J.); apayyanakkal@student.unimelb.edu.au (A.A.); minghao@student.unimelb.edu.au (M.H.Z.); 2 Department of Animal Science, School of Agriculture, College of Basic and Applied Sciences, University of Ghana, Legon, P.O. Box LG 226, Accra, Ghana

**Keywords:** milk production, panting scores, respiration rate, thermal camera

## Abstract

**Simple Summary:**

Heat stress (HS) is a major challenge for sustainable livestock production, which compromises animal welfare and performance during the hot summer months, leading to multibillion-dollar losses to the global livestock industries. In this study, we investigated the effect of summer heat events on lactating Holstein Friesian cows at the Robotic milking farm of the University of Melbourne in Victoria, Australia. We followed the cows during the entire summer period (December 2018–February 2019) and measured the effect of high temperature and humidity on physiological variables such as respiratory rate, panting scores and body temperature. All these parameters were significantly affected by high-temperature-humidity conditions with a significant drop in milk production. Most cows stopped grazing, sought shade, panted and spent more time at the watering points. These indicate that lactating dairy cows grazing summer pastures experience severe HS, compromising their welfare and leading to some changes in behaviour such as suspension of grazing and jumping into water troughs. However, the quantum of production losses, though significant, can be reduced by the provision of shade and water for the cows to cool down, as was observed in this study where the production losses were lower than previously reported in heat stress studies.

**Abstract:**

The objective of this study was to measure the impacts of summer heat events on physiological parameters (body temperature, respiratory rate and panting scores), grazing behaviour and production parameters of lactating Holstein Friesian cows managed on an Automated Robotic Dairy during Australian summer. The severity of heat stress was measured using Temperature-Humidity Index (THI) and impacts of different THIs—low (≤72), moderate (73–82) and high (≥83)—on physiological responses and production performance were measured. There was a highly significant (*p* ≤ 0.01) effect of THI on respiratory rate (66.7, 84.7 and 109.1/min), panting scores (1.4, 1.9 and 2.3) and average body temperature of cows (38.4, 39.4 and 41.5 °C), which increased as THI increased from low to moderate to high over the summer. Average milk production parameters were also significantly (*p* ≤ 0.01) affected by THI, such that daily milk production dropped by 14% from low to high THI, milk temperature and fat% increased by 3%, whilst protein% increased by 2%. The lactation stage of cow had no significant effect on physiological parameters but affected (*p* ≤ 0.05) average daily milk yield and milk solids. Highly significant (*p* ≤ 0.01) positive correlations were obtained between THI and milk temperature, fat% and protein% whilst the reverse was observed between THI and milk yield, feed intake and rumination time. Under moderate and high THI, most cows sought shade, spent more time around watering points and showed signs of distress (excessive salivation and open mouth panting). In view of the expected future increase in the frequency and severity of heat events, additional strategies including selection and breeding for thermotolerance and dietary interventions to improve resilience of cows need to be pursued.

## 1. Introduction

Demand for animal products is expected to increase due to human population growth, higher incomes, increased urbanization, and changes in dietary preferences [1,2,3,4] resulting in a need for increased production. At the same time, climate change poses a major threat to the viability and sustainability of livestock production systems [5]. Moderate to high ambient temperature and relative humidity compromises the ability of dairy cattle to maintain homeothermy and when the core body temperature is increased above the normal physiological level, heat stress (HS) occurs [6,7,8,9]. In dairy cattle, HS causes negative impacts on feed intake and milk production [10,11,12], growth and welfare [13], reproduction performance [7], health status and immune responses [5,14,15] resulting in a significant financial burden to the dairy industry [6,9,16,17,18,19]. In dairy cows, for instance, milk production has been reported to decline by 17–53% [11,20,21], feed intake by 35–48% [11,20] and low fertility was recorded in both first and second parities [18]. Additionally, conception rates from artificial insemination vary from 55% to less than 10% during the months of low and high temperatures and humidity, respectively [15]. A thermotolerant animal is one that maintains homeothermy under a high environmental heat load [22] or when the environmental temperature exceeds the species threshold. High-producing lactating cattle are most susceptible to HS on account of their relatively high increment of metabolic heat and continued selection for high production, which has negative impacts on cow welfare and productivity with variations in individual cow ability to tolerate this stress. Considerable variation in heat tolerance (HT) between and within cattle breeds has also been reported [8,23]. Therefore, the need to include a heat tolerance trait in the selection objective of dairy cattle populations [18,24] becomes more desirable. Furthermore, a lot of the previously reported studies have measured the impacts of heat stress on dairy cattle production using controlled climatic chambers simulating summer conditions or short-term heat events and there are limited studies reporting the actual production losses in dairy cattle grazing summer pastures. Therefore, this study was designed to measure physiological parameters (surface body temperature (SBT), respiratory rate (RR) and panting scores (PS)) and production parameters (daily milk yield, protein and fat content) of 120 lactating Holstein Friesian dairy cows grazing Australian summer pastures on an automatic robotic dairy

## 2. Materials and Methods

The experiment was approved by the University of Melbourne Faculty of Veterinary and Agricultural Science (FVAS) Animal Ethics Committee (AEC ID 1814645.1). A hundred and twenty (120) healthy Holstein Friesian cows in early to late lactation were used for the experiment which was carried out during the summer (ambient temperature ranged from 18–42 °C and relative humidity 25–75%) 2018/19 (December 2018–February 2019) at the University of Melbourne Dookie Campus

Robotic Dairy, which is in the Southern Hemisphere in the state of Victoria, Australia on latitude 36.4° S and longitude 145.7 °E (940 Dookie-Nalinga Road, Dookie College, VIC 3647, Australia). The dairy has a 43-hectare irrigated pasture with annual average rainfall of 540 mm. The multiparous (2–5th lactation) cows were blocked by stage of lactation as follows: early (≤120 days), mid (121–240 days) and late (>240 days) lactation. The average daily milk yield of the cows was 28.5kg (early lactation), 22.3 kg (mid lactation) and 19.9 kg (late lactation).

### 2.1. Data Collection

The period of data collection was the Australian summer period from 1st December 2018 to 28th February 2019. We collected weekly (on the day, predicted with ambient temperature >28 °C) field phenotypic data as well as daily milk production and quality data. Phenotypic data were collected between 14:00 and 18:00 h to take advantage of the highest temperatures of the day. Phenotypic data collected included RR, PS and SBT. Respiration rate was recorded via time in seconds taken for standing cows to make five flank movements (as the animal inhales and exhales with each breadth [25]) and calculated as respiration rate/minute. At the same time, the animals were observed for signs of drooling and/or open mouth panting and these data were used to determine the PSs of all cows (Table 1; [26]). Surface body temperature of cows was determined non-invasively using an infrared thermal camera. Infrared thermography (IRT) is a simple, effective, on-site, and non-invasive method that detects surface heat, which is emitted as infrared radiation and generates pictorial images without causing radiation exposure [27]. Additionally, Jorquera-Chavez et al. [28] and Hoffmann et al. [29] found acceptable correlations between temperature calculated from thermal infrared images and those collected from intravaginal loggers. Such automated phenotyping could provide temperature data in real time that would allow immediate intervention to prevent animal health related loses [30]. In this study, therefore, SBT of cows was determined using a thermal camera FLIR T1050sc (FLIR Systems, Wilsonville, OR, USA). The camera offers a thermal sensitivity of < 20 mK (NETD) and wide temperature range, with calibrations up to 2000 °C (–40 °C to +150 °C; +100 °C to +650 °C; +300 °C to +2000 °C). The accuracy of the camera is ±2 °C or ±2% of reading at 25 °C for temperatures up to 1200 °C, with emissivity of 0.985 [31]. Cows were photographed standing and under shady trees to minimize the effects of solar radiation and the distance between the photographer and the cows was kept constant between 3.5 and 4 m for all images taken at an angle of between 30°–40°. We analysed the images using the FLIR’s ResearchIR Max software [31] to obtain surface body temperature of five known body regions (eyes, forehead, flank, fore and hind udder) reported as proxies for body temperature [32,33]. Udder surface temperature for instance provides a reliable proxy of HS in cows on-farm [34]. The SBTs from the five body parts were thus averaged to obtain an estimate of the body temperature of the cows. Daily milk production, milk temperature, milk quality (somatic cell count, milk fat and protein %), cow weights and concentrate intake were collected automatically by the robotic milking machine (Lely Automatic Milking System), identifying individual cows via Radio Frequency Identification (RFID) ear tags. Additionally, each cow is fitted with a transponder (Qwes-HR, Lely) that contains a rumination monitor. The rumination monitor uses a microphone to detect chewing sounds and differentiates between eating and rumination time. Milk temperature (°C) was also recorded via the Lely Automatic milking system in use at the Dookie Dairy Farm. We recorded milk temperature on some selected hot days to gauge the effect of temperature humidity index (THI) on this parameter and also to see how it could be used as a proxy for body temperature in the future. Climatic data including daily minimum, average and maximum temperatures and relative humidity for the study period were downloaded for Dookie farm from the website of the Dookie Weather Meteorological station (http://weatherplus.ikcaldwell.com.au/).

In this study we calculated THI as follows

THI = (1.8 × T + 32) − (0.55 − 0.0055 × RH) × (1.8 × T − 26)

where T = temperature (°C); RH = relative humidity (%) [35].

For each day, we generated the minimum, average and maximum THIs. However, we used the maximum daily THI in our analysis [36] because milk yield, for instance, is more sensitive to the extreme values of the maximum THI relative to the daily average THI [37]. We then categorized the computed daily THIs into three groups, as follows: Low (THI ≤ 72), Medium (THI from 73 to 82) and High (THI ≥ 83).

### 2.2. Data Analysis

Data were analysed using one-way ANOVA with THI and lactation stage of cow as the main factors. We also computed Pearson correlation coefficients between THI and milk production parameters. We also computed various descriptive statistics of physiological as well as milk production and quality parameters and presented the mean and standard deviation (SD) to give an indication of the variability within our data [38,39]. All data analyses were carried out using the SPSS software (Version 26; [40]).

## 3. Results

Mean monthly maximum THI ranged from 76 to 81, with heat events reaching their peak in January 2019 with average THIs during the entire study period exceeding 72. Lactating cows respired significantly (*p* ≤ 0.05) faster and panted relatively more frequently under high THIs (Table 2). The average surface body temperature of lactating cows as measured by infrared thermometry also followed a similar trend, with significantly (*p* ≤ 0.05) higher SBTs recorded under high THI conditions. As expected, there was a decrease in average daily milk production per cow, with increasing THI down to 22 kg under high THI (≥83). On the other hand, milk temperature significantly increased with increasing THI. In terms of milk composition, both average fat and protein percentages were significantly higher at high THI. The cows, on average, also consumed less concentrate as THI increased (Table 3).

The stage of lactation of the cow had no significant (*p* > 0.05) effect on RR, PS and SBT, but significantly (*p* ≤ 0.05) affected average daily milk yield, fat %, protein % and concentrate feed intake (Table 3).

**Figure 1:**
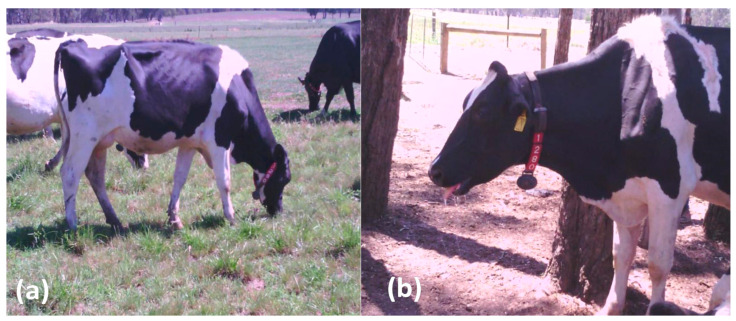
Effect of THI on cow behavior—same cow at THI = 70 (**a**) and THI = 84 (**b**).

Behaviour of the cows was also observed during the study period. Figure 1 shows images of the same dairy cow at the beginning of summer (THI = 70) and at the peak of summer (THI = 84). Clearly, the cow which was grazing comfortably in early summer (Figure 1a) is exhibiting signs of distress and open mouth panting during a heat event at the peak of summer (Figure 1b). In addition, most of the cows spent more time at the watering points and very little time grazing during heat events. At the peak of the heat events, the less thermotolerant cows spent the majority of time in the water troughs whilst other cows were struggling or waiting for the chance to get into available water troughs.

Highly significant (*p* ≤ 0.01) positive correlations were obtained between THI and RR, PS, SBT, milk temperature and protein (Table 4).

## 4. Discussion

The significant effect of THI on all measured three physiological parameters, namely respiratory rate (RR), panting score (PS) and surface body temperature (SBT) indicates a negative effect of HS on the experimental cows. Body temperature is regulated by the modulation of metabolic heat production and loss of heat from the body [41], and when an animal is unable to adequately dissipate excess endogenous heat to maintain homeothermy [42], HS occurs. The THI is a single value depicting the integrated effects of air temperature and humidity associated with the level of HS [43] and is used as a weather safety index to control and decrease HS-related losses [9,44]. Hot weather, as gauged by THI, is known to negatively affect cow performance primarily through reduced feed intake and some direct effects [45], as the reduction in feed intake does not account for all of the reduction in milk yield [11]. The THI has been found to be weakly to moderately related with core body temperature in lactating dairy cows exposed to HS [34]. Gonzalez-Rivas et al. [46] reported of a stronger relationship between rumen temperature and THI than between rectal temperature and THI in cattle maintained in a feedlot. In a review on measurements of peripheral and deep body temperature in cattle, Godyń et al. [47] provided a number of references, indicating that the core body temperatures of cattle could be measured via the ear canal, rectum and the vagina. In general, when THIs exceed 72 it means that cows are experiencing HS [26], and therefore dairy cows in the present study experienced HS throughout the three summer months (December 2018–February 2019). The relatively high average THI and low coefficient of variation recorded for January 2019 confirms it as the hottest period over the summer period. A decline in the thermal gradient between an animal and its surroundings due to high ambient temperature compromises the heat dissipation from an animal’s body and contributes to heat load [48]. Our results add to the evidence that HS is a real challenge for the dairy industry managing cows on pastures, particularly in the hot summer months. Heat stress has negative effects on cow welfare and productivity and projected future increases in temperature and humidity could lead to more significant negative implications for livestock productivity [49]. The exposure of high-milk-producing cows to heat stress conditions results in significant changes in their physiological and biochemical parameters, and therefore an assessment of the impact of THI on performance is important to mitigate any negative effects of heat stress [50]. All the physiological parameters measured in the present study were positively correlated with THI. Respiration rate is a reliable physiological parameter for predicting heat stress in dairy cattle [51] and has been found to increase with THI [52,53]. In this study, lactating cows thus respired faster and spent more time panting and trying to adjust to the HS conditions compared to cows under thermoneutral conditions. Panting sharply increases the loss of CO_2_ via pulmonary ventilation, reducing the blood concentration of carbonic acid and upsetting the critical balance of carbonic acid and bicarbonate necessary to maintain blood pH, resulting in respiratory alkalosis [10]. This situation poses further challenges to the physiology and welfare of dairy cows, as compensation for the respiration alkalosis involves increased urinary bicarbonate excretion, leading to a decline in blood bicarbonate concentration which, together with losses of bicarbonate with excessive salivation, results in rumen acidosis [10,54]. In other studies, respiration rates were found to be significantly higher (*p* < 0.01) in the afternoon than in the morning and this was attributed to changes in ambient temperature and relative humidity [55]. In a study on the daily rumination time of dairy cows under heat stress, Muschner-Siemens et al. [56] concluded that rectal temperature (RT) is a useful tool to detect HS in dairy cattle. Gantner et al. [57] also reported that high-producing Holstein cows had a greater increase in RT than low-producing cows and the Simmental breed was more resistant to HS in terms of changes in daily milk production and somatic cell counts. In line with the findings of the present study, increases in body temperature and RR have also been found to be negatively correlated with overall milk yield [46,58]. Under hot conditions (temperature 24–39 °C and relative humidity 32–60%) a 1 °C increase in ambient temperature can increase respiration rate from 2.8 to 3.3 breaths per minute [59] and all these physiological responses to heat are the cows’ coping strategies [19].

In the present study, the significant effect of stage of lactation of the cow moderated the effect of THI on average daily milk yield (ADMY), yet the lowest ADMY was obtained under the highest THI conditions. In terms of milk quality, we observed a trend of high milk solids (fat and protein %) with increasing THI confirming the significant (*p* ≤ 0.05) negative correlation between ADMY and milk solids (Table 4). Although early lactating cows had a significantly (*p* ≤ 0.05) higher ADMY and correspondingly concentrate feed intake, their milk solids were significantly (*p* ≤ 0.05) lower, as one would expect.

The Dookie Robotic Dairy Farm is a modern farm with some heat mitigating facilities to ensure cow comfort during heat events. The farm has the provision of shade (trees) and sprinklers and misting fans have been installed in the robotic milking parlour for the cows during the summer months. In spite of all these facilities, we observed cows suffering from HS, indicating that current environmental mitigating strategies on majority dairy farms may not be adequate to completely solve the problem of HS during summer. This also confirms earlier findings that, even on well cooled dairies or in temperate areas, heat stress decreases milk yield by 10–15% and on non-cooled management systems, milk yield can decrease by as much as 40–50% [8,17]. This can be partially attributed to the reduction in feed intake and changes in postabsorptive metabolism [21] as a result of the HS, and plausibly due to the significant (*p* ≤ 0.05) negative association between THI and milk production. The magnitude of reduction in milk yield by heat stress is influenced by different mechanisms at different stages of lactation and the mammary glands of early-, mid-, and late-lactation cows may respond differently to heat stress [60]. The present findings also corroborate previous research, which associates high THI conditions negatively with forage intake, milking frequency and milk yield in pasture-based automatic milking systems [7,42,44,61,62]. Brugemann et al. [37] also reported a substantial decline in daily milk yield for THI > 60, whilst Bohmanova et al. [44] found that HS in dairy cows mostly occurs in THI ≥ 70. A more recent study suggested that the heat load threshold for lactating cows was THI = 70 when cows were standing and THI = 65 when cows were lying down [53]. However, it is important to mention that the duration of decline in milk yield also depends on the month of the measurement and thermal conditions in the preceding month [63]. Byrant et al. [64] reported that hot conditions were associated with reductions in milk and milk solid yields, and fat and protein concentrations in three breeds of dairy cattle (Holstein Friesians, New Zealand Jerseys and crosses between the two) in New Zealand with better tolerance in the Jersey cows. Nasr et al. [65] reported that daily milk yield and composition (fat%, protein %, yielded fat, yielded protein and the percentage of lactose) were higher under THI ≤ 72 (31.91 kg, 3.91%, 3.22%, 418 kg, 349 kg and 4.20%, respectively) when compared with THI ≥ 83. Heat stress has also been reported to adversely affect production in the lactating dairy cows, and there is often a delay before a return to normal feed intake and milk yield following the heat challenge, indicating a period of metabolic recovery [20]. It has also been reported that a decrease in cow milking performance also depends on the severity of the heat wave and the length of the heat during the preceding periods [63]. Bouraoui et al. [56] explained that a part of the adverse effects of HS on milk production could be attributed to reduced nutrient intake and decreased nutrient uptake by the portal drained viscera of the cow. The redistribution of blood stream away from gastrointestinal tract to peripheral tissues for cooling functions may alter nutrient absorption and metabolism, and contribute to lower milk yield during hot weather.

Dairy cows undergo a series of complex responses at the molecular and cellular level which are essential for cell survival during HS and behavioural and metabolic responses to reduce exposure to direct heat and increase avenues to lose heat from the body [7,66]. Respiration rate and body core temperature have been recommended as suitable parameters to assess individual heat loads of dairy cows at an early stage, whereas feeding behaviour may depend on the physiological stage of the cow [66]. In mammals, exposure to hypothermia or hyperthermia has been related to morphological and physiological alterations [67]. In the present study, we observed that almost all the cows sought shade as the heat intensity increased, indicating obvious distress and a search for shelter from the direct sun. This is in line with previous findings that, even in temperate areas, cattle may suffer from HS when they are grazing pasture in summer, and the provision of shade helps to reduce such stress [68,69]. Therefore, it is important to mitigate the stressful effects of hot climate on dairy cows by protecting them from direct and indirect solar radiation [10]. On the other hand, HS mitigation should be factored into barn construction for intensively bred dairy cattle to achieve optimum insulation, and reasonable orientation for ventilation as well as bedding material [70]. Modification of the environment around the animal (provision of shade, fans, sprinklers, mists) and nutritional interventions (dietary antioxidant supplementation) may be immediate methods to alleviate HS but these are expensive and not always economically viable for producers [17]. The increased frequency of shade seeking, cows spending more time at the watering points, panting and eating less all indicate that cows were affected by the heat events and the environmental strategies in place at the farm may not have been adequate to totally ease HS. This herd comprises Holstein Friesian cattle which have been traditionally selected for higher milk yield, leading to increased metabolic heat production, possibly making them more susceptible to HS [8] due to the antagonism between productivity and HT, confirming the hypothesis that, with increasing global warming, HS will become worse in the future [10]. Therefore, selection for HT is timely to prevent further deterioration in tolerance of HS [71].

The current study found that dairy cow performance was better in most of the investigated parameters at a low THI than those in high THI. Thus, in agreement with previous studies, a detrimental effect of THI on both dairy cow welfare and farm economic returns was demonstrated under natural Australian summer conditions. Given this scenario, the need to explore other options to sustain dairy cattle productivity in warm climates becomes increasingly attractive, especially the need to explore genetic variation in heat stress. Lee et al. [72] concluded that genetic evaluation using THI could be applied to select bulls for thermotolerance and recommended that sire rankings should be changed to incorporate the effects of high temperature and humidity. Additionally, heat tolerance in dairy cattle could be improved using genomic selection [73]. Garner et al. [8] demonstrated that dairy cattle were predicted by genomic breeding values to be heat tolerant, have less of a decline in milk production, and reduced increases in core body temperature during a simulated heat wave event in comparison to cows predicted to be heat-susceptible. Thus, genomic selection for heat tolerance could increase the resilience and welfare of dairy herds and the productivity of dairy farming in a future with increased incidence and duration of heat stress events.

## 5. Conclusions

Heat stress has serious negative impacts on lactating dairy cows during the hot summer months and therefore poses a challenge for the sustainability of the dairy industry in temperate regions with the increasing frequency and severity of high heat events in response to climate change. The findings of this study clearly indicate that lactating dairy cows grazing summer pastures experience HS and this reduces their production and affects their comfort, as indicated by increased respiration and panting. It is also evident that no single strategy would be enough to mitigate HS but that a combination of mitigation strategies such as the provision of shade and water, nutritional interventions, and selection for heat tolerance needs to be adopted on farms to support sustainable dairy operations under a changing climate.

## Figures and Tables

**Table 1:** Scale used for respiratory rates and panting scores (adapted from Gaughan et al. [26]).

Breathing Condition	Panting Score (PS)	Respiratory Rate (RR) (Breaths/Minute)
Normal panting—normal (difficult to see chest movement)	0	≤40
Slight panting—mouth closed; no drool or foam, easy to see chest movement	1	40–70
Fast panting—drool or foam present; no open mouth panting	2	70–120
Like panting score 2 but with occasional open mouth, tongue not extended	2.5	70–120
Open mouth with some drooling; neck extended and head usually up	3	120–160
Like panting score 3 but with tongue protruded slightly, occasionally fully extended for short periods with excessive drooling	3.5	120–160
Open mouth with tongue fully extended for prolonged periods and excessive drooling; neck extended and head up.	4	>160
As for 4 but head held down; cattle ‘breath’ from flank; drooling may cease	4.5	Variable—RR may decrease

**Table 2:** Effect of THI (Temperature-Humidity Index) on physiological and milk parameters (Mean ± SD) of dairy cows.

Parameter	THI		
	≤72	73–82	≥83
Sample size (n)	518	1175	666
Respiratory (rate/min)	66.0 ^c^ ± 18.8	81.8 ^b^ ± 21.4	113.1 ^a^ ± 31.5
Panting score	1.38 ^c^ ± 0.63	1.87 ^b^ ± 0.61	2.42 ^a^ ± 0.64
Surface body temperature (°C)	37.8 ^c^ ± 1.86	39.5 ^b^ ± 2.07	41.7 ^a^ ± 1.08
Daily milk production (kg)	23.1 ± 7.59 ^ab^	23.5 ± 6.11 ^a^	22.2 ± 5.4 ^b^
Milk temperature (°C)	38.7 ± 0.75 ^c^	39.7 ± 0.74 ^b^	40.0 ± 1.03 ^a^
Milk fat (%)	4.25 ± 0.59	4.21 ± 0.74	4.34 ± 0.78
Milk protein (%)	3.05 ± 0.27 ^b^	3.10 ± 0.22 ^ab^	3.14 ± 0.23 ^a^
Daily concentrate intake (kg)	5.68 ± 1.69 ^b^	5.06 ± 1.78 ^a^	5.13 ± 1.79 ^a^

^a,b,c^ Within rows means with different superscripts differ significantly (*p* ≤ 0.05).

**Table 3:** Effect of lactation stage on physiological and milk parameters (Mean ± SD) of dairy cows exposed to THI from 70 to 84.

Parameter	Lactation Stage of Cow		
	Early (≤120 days)	Mid (121–240 days)	Late (≥240 days)
Sample size (n)	637	1080	642
Respiratory rate/min	101.1 ± 32.6	97.8 ± 33.8	104.6 ± 32.8
Panting score	2.19 ± 0.69	2.12 ± 0.75	2.26 ± 0.71
Surface body temperature (°C)	40.8 ± 1.88	40.5 ± 2.21	41.0 ± 1.77
Daily milk production (kg)	28.5 ^a^ ± 6.15	22.3 ^b^ ± 5.67	19.9 ^c^ ± 3.70
Milk temperature (°C)	39.8 ± 0.96	39.7 ± 1.08	39.9 ± 0.9
Milk fat %	3.67 ^c^ ± 0.41	4.20 ^b^ ± 0.69	4.82 ^a^ ± 0.67
Milk protein %	3.03 ^b^ ± 0.17	3.10 ^ab^ ± 0.22	3.21 ^a^ ± 0.26
Daily concentrate intake (kg)	5.44 ^a^ ± 1.74	5.34 ^a^ ± 1.70	4.72 ^b^ ± 1.89
Somatic cell count (SCC)	189.2 ± 24.1	151.2 ± 14.6	213.7 ± 29.2

^a,b,c^ Within rows means with different superscripts are significantly (*p* ≤ 0.05) different.

**Table 4:** Pearson correlation coefficients between THI and milk production and quality parameters.

	THI ^#^	RR	PS	SBT	ADMY	MT	Fat %	Protein%	SCC
RR	0.54 **								
PS	0.50 **	0.90 **							
SBT	0.66 **	0.50 **	0.46 **						
DMP	−0.08	0.01	0.05	−0.07					
MT	0.39 **	0.30 **	0.29 **	0.23 **	0.09 *				
Fat%	0.08	0.09 *	0.09 *	0.03	−0.40 **	0.07			
Protein%	0.15 **	0.10 *	0.09 *	0.001	−0.29 **	0.05	0.53 **		
SCC	0.01	−0.02	−0.02	−0.07	−0.15 **	0.05	0.20 **	0.19 **	
CI	−0.04	−0.02	−0.05	−0.16 **	0.38 **	0.003	−0.27 **	−0.13 **	−0.10 *

** *p* ≤ 0.01; * *p* ≤ 0.05. **^#^** THI—temperature–humidity index; RR—respiratory rate; PS—panting score; SBT—surface body temperature; ADMP—average daily milk yield; MT—milk temperature; SCC—somatic cell count.
